# Micro-Electro-Mechanical Systems Microphones: A Brief Review Emphasizing Recent Advances in Audible Spectrum Applications

**DOI:** 10.3390/mi15030352

**Published:** 2024-02-29

**Authors:** Zhuoyue Zheng, Chen Wang, Linlin Wang, Zeyu Ji, Xiaoxiao Song, Pui-In Mak, Huafeng Liu, Yuan Wang

**Affiliations:** 1 Institute of Microelectronics, State Key Laboratory of Analog and Mixed-Signal VLSI, University of Macau, Macau 999078, China; yc37907@um.edu.mo (Z.Z.); mc36153@um.edu.mo (Z.J.); pimak@um.edu.mo (P.-I.M.); 2 Department of Electrical Engineering-MNS, University of Leuven, 3000 Leuven, Belgium; chen.wang@kuleuven.be (C.W.); linlin.wang@kuleuven.be (L.W.); 3 School of Physics and Information Engineering, Jiangsu Second Normal University, Nanjing 210013, China; 4 MOE Key Laboratory of Fundamental Physical Quantities Measurement, PGMF and School of Physics, Huazhong University of Science and Technology, Wuhan 430074, China

**Keywords:** MEMS microphone, environmental noise-cancelling, directional microphone, capacitive microphone, piezoelectric microphone

## Abstract

The MEMS microphone is a representative device among the MEMS family, which has attracted substantial research interest, and those tailored for human voice have earned distinct success in commercialization. Although sustained development persists, challenges such as residual stress, environmental noise, and structural innovation are posed. To collect and summarize the recent advances in this subject, this paper presents a concise review concerning the transduction mechanism, diverse mechanical structure topologies, and effective methods of noise reduction for high-performance MEMS microphones with a dynamic range akin to the audible spectrum, aiming to provide a comprehensive and adequate analysis of this scope.

## 1. Introduction

The microphone, a device capable of sensing acoustic vibrations and transducing them into electrical signals, has experienced an exponential evolution since its emergence. Extending from its origins, it has expanded into various domains including commercial communication, medical applications, industrial usage, and both surveillance and military sectors.

The inception of the electret condenser microphone (ECM) marked a pivotal milestone, pioneered by Gerhard Sessler and James West in the early 1980s [1]. Subsequently, the advancement of MEMS (Micro-Electro-Mechanical Systems) technology coupled with strides in materials science drove a shift in this area, enabling the possibility of the miniaturization of macro-counterparts. This revolution not only reduced the size factor of the devices but also propelled substantial innovations in the operational mechanisms of microphones, offering newfound perspectives and increased design flexibility [2].

The merits of miniaturization are distinct, particularly in rendering a better noise performance, reduced power consumption, and multifunctional capabilities for electronic devices. As such, MEMS microphones are prevailing in consumer electronics like smartphones, tablets, wearables, computers, automobiles, and IoT devices, and are expected to see a consecutive growth in demand. According to a report from Maximize Market Research, MEMS Microphones Market is worth USD 1.82 billion in 2022 and is expected to hit USD 3.97 billion by 2029 at a CAGR of 11.8 percent [3].

Presently, the most economically competitive transduction mechanisms of MEMS microphones in use encompass capacitive, piezoresistive [4], and piezoelectric [5] types. Among these, capacitive microphones stand out for offering relatively higher signal strength, reaching up to hundreds of microvolts. However, they struggle with additional power consumption and exhibit a relatively high sensitivity to environmental factors such as dust and humidity, a complex manufacturing process, and could be more susceptible to electromagnetic interference. Despite these drawbacks, the simplicity of their structural design and compatibility with CMOS technology allows them to retain the leading position of capacitive MEMS microphones in the commercial market. In contrast, although piezoresistive microphones provide a wider dynamic range, they struggle with limitations regarding sensitivity and power consumption.

Utilizing piezoelectric-based materials to facilitate acoustic sensing presents the advantage of passive devices without additional input power, rendering them well-suited for portable applications. Furthermore, their robust design, making them less susceptible to environmental factors. requires simpler manufacturing process compared to capacitive MEMS microphones. However, achieving a comparable performance with capacitive microphones has posed a persistent challenge, in particular, concerning CMOS-compatible materials such as aluminum nitride (AlN) or zinc oxide (ZnO) [6]. In addition, the operational range of piezoelectric MEMS microphones is contingent upon the material used, indicating a promising potential in the future market. Collectively, microphone applications take into account a delicate balance between the demands of various usage scenarios, process intricacies, reliability, and environmental sustainability.

It has been decades since the very first microphone was employed in practical use, witnessing the proclamation of numerous brilliant designs and subsequent reviews in the fields. In 2018, Ishfaque et al. [7] launched a review paper mainly focused on directional microphones inspired by the parasitic fly called Ormia Ochrasia. In 2019, Shah et al. [8] presented a comprehensive review covering the diverse transduction mechanisms of microphones and summarizing data from academic papers and commercial products. Then, in 2020, Zawawi et al. [9] offered a review that emphasized capacitive microphones. Subsequently, in 2022, Kumar et al. [10] presented a comprehensive literature on piezoelectric microphones, along with the fabrication processes and methodologies used for experimentations. Thereafter, Gemelli et al. [11] summarized some of the state-of-the-art scenarios, presenting the recent performance advances.

Having mentioned the many review works, a specific article reviewing microphones to voice applications is yet to be conducted. Microphones used for human voice signals have rigorous requirements for input signal sound pressure-level characteristics and sensitivity. To fill the gap in reviewing such a subject, this article will orient on high-performance MEMS microphones structured specifically for sensing human voice signals.

Microphones tailored to human voice applications are typically designed with a dynamic range akin to the audible spectrum of the human ear, approximately 20 Hz to 20,000 Hz, and exhibit peak sensitivity within the presence range of 3 kHz to 7 kHz. Considering the fact that a typical conversation has a sound pressure level (SPL) of about 60 dB, and the highest recorded human voice reaching approximately 135 dB (at a distance of 1 inch from the mouth), with loud vocals typically peaking around 115 dB to 120 dB SPL, high-fidelity microphones must ensure a flat and sensitive response within the pertinent frequency band. In accordance with this, a tradeoff between dynamic range and maximum input sound pressure level is inevitable.

For the applications of microphones in daily scenarios, environmental noise often presents a relatively consistent challenge. Thereby, augmenting the signal-to-noise ratio (SNR) by enhancing the mechanical and electrical sensitivity of microphones, enlarging the signal amplification, and adopting signal enhancement techniques are effective strategies for noise reduction. Following this concept, the first part of this review article will highlight the state-of-the-art methods for boosting sensitivity and SNR. To complement this, utilizing mechanical structures and algorithms to suppress environmental noise are alternative approaches to noise reduction. The second part of this article will focus on providing solutions and analysis for reducing environmental noise through these means. Finally, this article will conclude by summarizing the discussed aspects and envisioning solid directions for further exploration and consideration.

For optimal microphone performance, a meticulous consideration of cavity dimensions and package design is imperative. The size of the back cavity plays a pivotal role in influencing the device’s low-frequency response. When employing lumped element modeling, the cavity is modeled as an acoustic mass and compliance, forming an analogous series LC circuit. The strategic adjustment of the back cavity size holds the potential to augment low-frequency signal strength. However, the realization of the back cavity typically involves processes like deep reactive ion etching (DRIE) and reactive ion etching (RIE). Beyond the release of membrane or beam structures, these processes are intricately linked with the reliability observed during sample chip testing. Remarkably, papers spotlighting vibrational structures seldom delve into a deliberate characterization of the impact of back cavity dimensions on device performance. And, it is essential to note that most of the microphone structures discussed in this article have not undergone industrialization, and have only been characterized in a laboratory setting; therefore, the packaging process is not the primary concern in this context. Thence, the details related to cavity and packaging have not been extensively elucidated in this paper.

## 2. MEMS Microphone Transduction Mechanism

### 2.1. Capacitive Transducer

**Figure 1:**
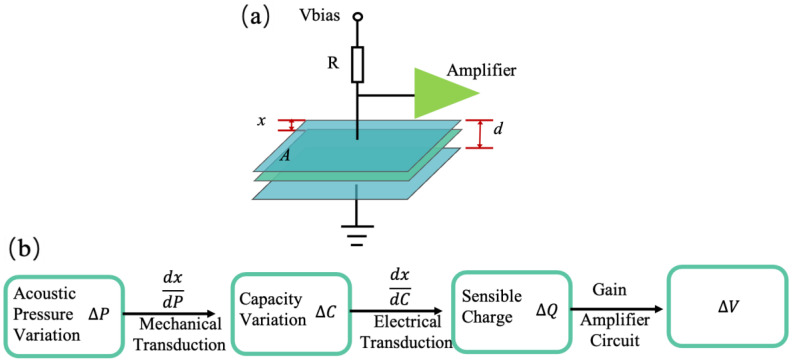
(**a**) Schematic diagram of capacitive sensing principle. (**b**) Working principle of capacitive microphone.

In general, the capacitive sensing model applied to the microphone is simplified and depicted in Figure 1a. The sensitivity of a capacitive microphone consists of electrical sensitivity and mechanical sensitivity, and its working principle is illustrated in Figure 1b.

The electrical sensitivity and the mechanical sensitivity can be expressed by:

(1) $$S_{el}=\left|\frac{dV}{dx}\right|=\left|\frac{dQ}{dxC}\right|=\frac{V_{bias}}{d},$$

(2) $$S_{mech}=\left|\frac{dx}{dp}\right|=C_{A}\times A=C_{s},$$

Thus, the sensitivity of the microphone can be expressed as

(3) $$S=S_{el}S_{mech}=\frac{V_{bias}C_{A}A}{d},$$

where *x* is the membrane deflection and *p* stands for sound pressure, *C_A_* is the acoustic compliance, *A* is the area subject to sound pressure, and *C_s_* is the specific compliance in m/Pa. A high bias voltage, a narrow capacitive gap, and a transducer with a high compliance are pivotal factors for achieving high sensitivity in microphones. However, in low-voltage applications, reducing the bias voltage becomes advantageous. Strategies for raising bias voltage encompass increasing the total capacitance area of transduction capacitance, although this is controversial in smaller-sized designs. Another approach involves decreasing the spring constant of the diaphragm, a feat achievable through low-stiffness structures.

#### 2.1.1. Expanding the Effective Diaphragm Area

To better estimate the performance between domains, an effective area is typically used in the contents of capacitive sensing mechanics, instead of a geometric one labeled ‘*A*’. Effective area $A_{eff}$ represents a distributed system as the network of lumped elements, denoted by [12]:

(4) $$A_{eff}\times y_{c}=\int_{0}^{a}y(r)2\pi rdr,$$

and *y*(*r*) is the diaphragm deflection as a function of this radius, which is determined by a boundary condition. Normally, we use effective area coefficients to describe the relationship between *A* and *A_eff_*:

(5) $$\beta =\frac{A_{eff}}{A_{d}}=\frac{\int_{0}^{a}y(r)2\pi rdr}{A_{d}y_{c}},$$

Thus, a larger β may imply a more piston-like motion between two electrodes of the capacitor, and a larger “effective area” implies higher trans-efficiency. The foremost method to increase the effective membrane area is through employing a dual-backplate or dual-diaphragm structure. Additionally, utilizing a differential output scheme proves advantages for the signal-to-noise ratio (SNR), Active Open-Loop (AOL), and common-mode noise reduction, thereby enhancing the overall performance [13].

**Figure 2:**
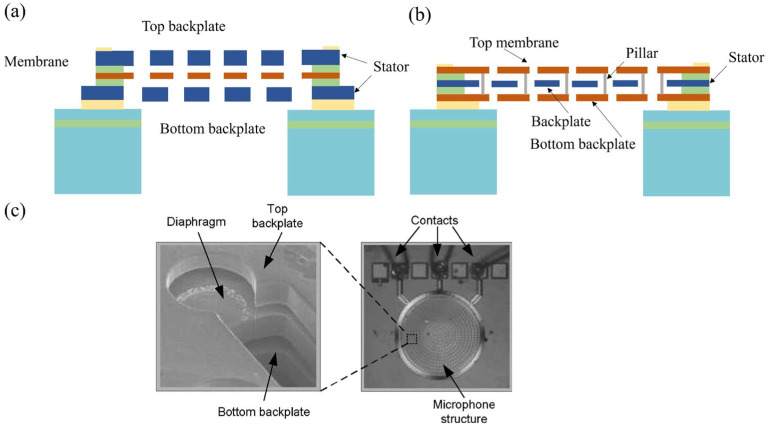
(**a**) Schematic cross-sectional view of the dual-backplate microphone. (**b**) Schematic cross-sectional view of dual-membrane microphone. (**c**) Close-up view of the microphone structure and wire-bond connections (Reprinted with permission from Ref. [16]).

The earliest proposal for a dual-backplate structure by Rombach et al. The schematic cross-sectional view of the dual-backplate microphone is similar as shown in Figure 2a. They introduced a front and backplate configuration that resulted in a notably stiffer acoustic structure. This design showcased a 10 dB increase in sound pressure level (SPL) compared to conventional microphones. Martin et al. [14,15,16] achieved a simplification of the process building upon Rombach’s research, as shown in Figure 2c, leading to reduced manufacturing costs. Simultaneously, modifications to the membrane size tailored for aero-acoustic applications enable a broader dynamic range. Theoretically, with the assumption of identical displacement conditions, such a design can double the mechanical sensitivity.

However, if the absolute membrane area differs from the effective membrane area, the former will cause unwanted overlaps between structures and inadvertently increase the total parasitic capacitance. On the other hand, experimental data reveals that the signals from the two backplates are not consistent. According to Rombach, only 17% of the upper backplate’s capacitance is parasitic and does not contribute to the signal, while the lower backplate has a parasitic portion of 63%. Subsequently, other researchers have explored structural designs involving the membrane to augment the spring-anchor system [17]. Furthermore, one has employed a multi-objective optimization procedure aided by the NSGA-II algorithm [18], but these methods did not exhibit remarkable improvements in the sensitivity of the dual-backplate configuration.

Apart from the dual backplate, Sant et al. [19] developed a sealed-dual membrane (SDM) MEMS transducer as shown in Figure 2b. The design is paired with the latest generation of digital read-out ASIC.

In general, for dual-backplate or conventional microphones, a compromise sits in between the acoustic resistance of the backplate perforations which causes Johnson noise and the loss of the front-facing area due to the backplate perforations. Sant introduced modifications to the dual-backplate topology by employing two flexible, non-perforated membranes, achieving a sealed construction in a vacuum. This setup allows for reduced pressure within the air gaps, enabling the sensor to operate in a low-viscosity ambient environment. Apart from the advantages of differential microphone systems, dual-membrane configurations also offer significant inherent environmental resilience. However, the primary drawback lies in the complexity of the vacuum-sealing process and pillar formation. Despite challenges in fabrication, the dual-membrane structure remains highly competitive due to the superior performance it can offer and has been implemented by Infineon in their XENSIV series, demonstrating its competitiveness in practical applications. In the dual-membrane structure, when the sealed membranes deflect in the upward and downward directions, the vertical edge of the first clamping layer extending beyond that of the second clamping layer can create a hot spot or notch effect. In a patent released by Infineon [20], they have significantly addressed these deflections. They achieved this by employing layers with slow etching rates and fast etching rates to form anchor structures that incline from the fixed backplate towards the thin film, mitigating the occurrence of hot spots or notch effects.

In a patent released by the company Knowles [21] at the end of 2023, a dual-membrane design is mentioned. Unlike the approach implemented by Infineon, this design deviates in that it does not employ a vacuum-based dual-membrane bonding process. Simultaneously, the pillars connecting the two membranes are divided into three regions based on 30% and 70% of the radius, with each region containing pillars of varying cross-sectional areas.

#### 2.1.2. Diaphragm Compliance

**Figure 3:**
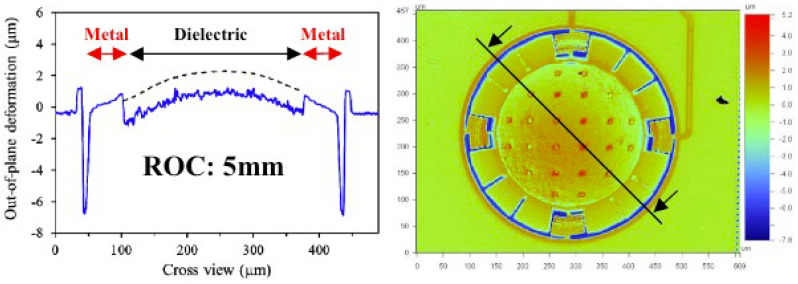
Measurements and photos of structure deformation of the microphone diaphragm after deposition process (Reprinted with permission from Ref. [23]).

The deflect magnitude of the diaphragm is determined by its effective spring constant *k* to measure of how resistant a membrane is to deformation when subjected to a force, expressed as

(6) $$k=8\pi \delta_{d}t_{d},$$

where $\delta_{d}$ is tensile stress in Pascal (Pa), and *t_d_* is the diaphragm thickness in meters. Regarding the compliance mentioned in Equation (2), it is the reciprocal of *k* and measures the ease with which a membrane can be deformed. Spring constant (*k*) and compliance (*C*) are inversely proportional to each other, thus an elevation of the parameter *k* leads to a reduction in *C_A_*, thereby diminishing sensitivity. The stress within silicon diaphragms is typically influenced by the deposition process, often causing warping in thin films. For sound pressure levels (typically between 20 μPa to 20 Pa), the impact of residual stress on mechanical sensitivity cannot be neglected [22]. The deformation of the microphone diaphragm caused by residual stress is shown in Figure 3.

Bulk micromachining and surface micromachining hitherto are the main strings of the capacitive microphone fabrication process, wherein the initial stress can hardly be predicted and maintained stably owing to the fluctuations in the manufacturing environment and the parameter deviations of the instruments. So, some structure-wise designs like corrugated membranes and spring arms are invested in to reduce the initial stress, hence preserving compliance. Additionally, as suggested in [24], using SOI (Si on insulator) wafers for fabrication can naturally prevent the residual stress problem for MEMS microphones.

It has first been shown by Jerman [25] that corrugated diaphragms can be made accurately in silicon using micromachining techniques. The corrugated capacitive microphone can promise a flat center zone to maximize the effective area and has been calculated to tune the stress factor of 1000–10,000. Therefore, one of the applications of corrugated diaphragms is the decoupling of a mechanical sensor from the influence of temperature changes and packaging stress [26]. Accordingly, the depth and number of corrugated rings should be well devised because mechanical sensitivity is not monotonically related to those. On the contrary, the bending stiffness of the corrugations can be the dominant factor if the corrugation is too deep. In a patent released by Infineon in 2023 [27], a highly sensitive microphone is achieved through the utilization of a porous backplate and a corrugated diaphragm. In addition to introducing corrugations on a circular diaphragm, they innovatively proposed placing corrugations on a spring-like beam structure, thereby further enhancing the compliance of the membrane structure.

**Figure 4:**
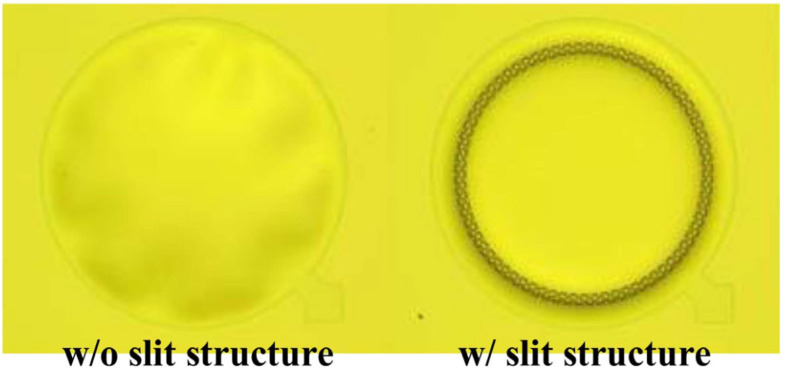
Optical images of the membrane with or without slit structure (Reprinted with permission from Ref. [23]).

In some circumstances, slits can occur in the diaphragm structure if the corrugations are made deeper, as per the findings of Yoo et al. A membrane with slit edges significantly increases the compliance of the membrane [23]. The optical image in Figure 4. depicts the membrane is shaded without slits, indicating an irregular surface.

**Figure 5:**
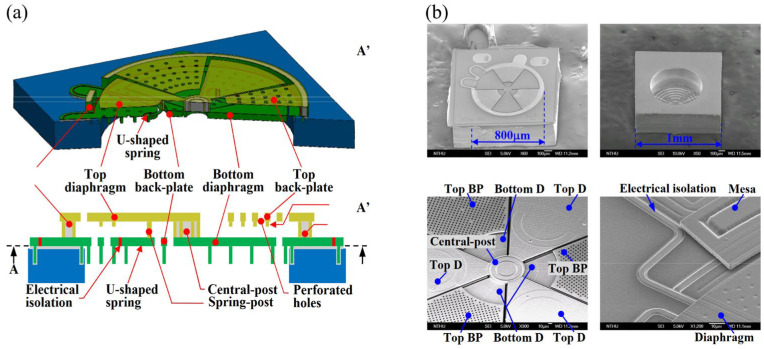
(**a**) Schematic design and AA’ cross-section view of sliced microphone. (**b**) Front-side (top-left) and back-side (top-left) views; zoom-in of diaphragm (bottom-left and bottom-right) (Reprinted with permission from Ref. [28]).

Expanding on the concept of slits, sliced membranes offer a further extension in functionality. The work proposed by the Lo team [28] gives an idea of combining of sliced membrane for residual stress releasing and a single sacrificial layer to define differential sensing gaps, resulting in an extra 6dB boost in SNR. As schematic design shown in Figure 5a. The mesas surrounding the diaphragm promise the reduction of low-frequency acoustic loss and broaden the bandwidth. They introduced the idea of involving a rigid membrane, via-posts, and variable gap achieved through a spring mechanism. In their work, the spring structures were designed in both U-shaped [28] and V-shaped [29] configurations, as illustrated in Figure 5b, intending to provide improved acoustic compliance.

It has also been found that flexible springs are employed to attach the diaphragm, aiming to reduce residual stress, tune the spring constant (K), and further augment the effective area. Numerous studies focus on designing spring-like arms to create a spring-mass oscillator system or employing suspension structures. This allows the membrane’s vibration to achieve a piston-like deformation, proven to effectively enhance sensitivity. Noteworthy designs that combine spring mechanisms with other factors are elaborated in what follows.

**Figure 6:**
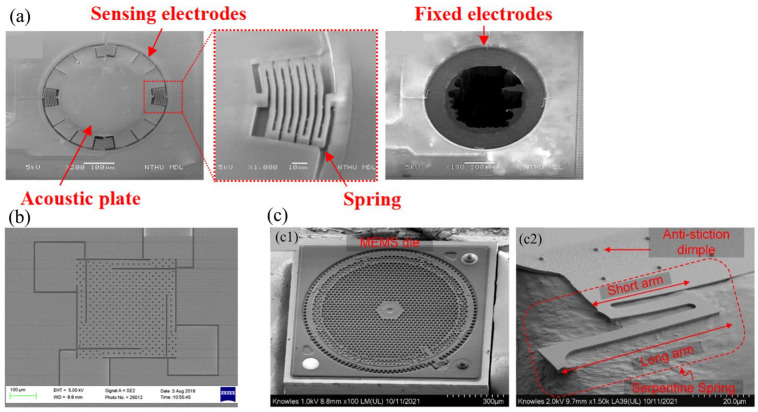
The spring designs. (**a**) SEM of a spring structure of “no backplate” microphone (Reprinted with permission from Ref. [30]). (**b**) SEM of the out-of-plane area-changing sensing architecture (Reprinted with permission from Ref. [31]). (**c**) SEM of a perforated backplate and serpentine spring design. (c1) Top view of the MEMS microphone die, (c2) serpentine spring design (Reprinted with permission from Ref. [12]).

Mao [30] employed a spring structure to achieve a “no-backplate” capacitive MEMS microphone design. The design is shown in Figure 6a. The sensing electrode is positioned at the edge of the acoustic membrane, while the spring facilitates the suspension of the membrane structure. As can be seen from the as-fabricated device, the backplate features ring-shaped and membrane-sized vent holes. Expanding on this design, Lo [24] enhanced the process based on Mao’s work. They utilized Silicon-On-Insulator (SOI) technology to create a linear out-of-plane area-changing sensing architecture, aiming to achieve lower residual stresses and improved temperature stability.

Weigold et al. [32] and Ganji et al. [31] provided a solution for combining the SOI process with a spring-like arm. The proposed structures are shown in Figure 6b. Theoretically, spring-shape arms decrease diaphragm stiffness and air damping while SOI wafers can further vice the residual stress during the deposition process, the cooperation of which can yield high compliance, as well as spare fabrication time and cost. Unfortunately, experimental results have not shown a significant enhancement in the response, possibly due to the intrinsic defect in the SOI process.

As shown in Figure 6c, Shubham et al. [12] presented the design of a semi-constrained polysilicon diaphragm with flexible springs and stopper structures, wherein the diaphragm is simply supported with a center and eight peripheral protrusions extending from the backplate. Protrusions serve as stopper structures to further increase the effective area, linearity, and sensitivity of the diaphragm under an applied bias voltage.

Furthermore, at the end of the year 2022, the Knowles Company [33] also unveiled a patent wherein a stiffening member is introduced along the edges of the membrane. The stiffening member comprises multiple fingers extending inward from the perimeter of an aperture defined by the transducer substrate. The stiffening members are configured to reduce stress in the diaphragm near the anchoring points for the diaphragm due to a thickness change, for instance at an electrode boundary. By reducing the maximum stress in the diaphragm, the stiffening members can, advantageously, increase the pressures and loads that can be tolerated by the MEMS transducer.

**Figure 7:**
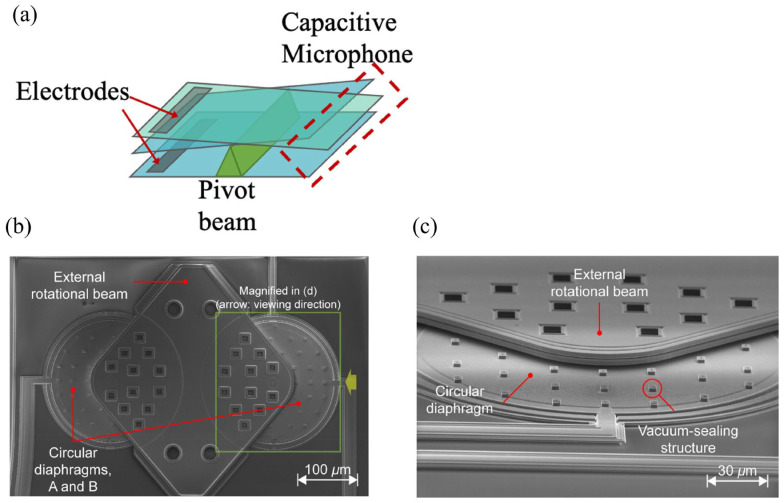
(**a**) Schematic rotate capacitive microphone. (**b**) Micrograph of the rotational microphone. (**c**) The magnified view of the structures on the rotational microphone (Reprinted with permission from Ref. [36]).

Further, Touse and Liu have adjusted stiffness by employing a teetertotter-style microphone, and its schematic is shown in Figure 7a. The proposed structure ideally offers extremely low stiffness against rotations about the pivot beam [34,35]. To be more specific, the working principle of a teetertotter-style microphone involves connecting two conventional parallel plate capacitive structures via a beam that rotates about a pivot or supporting hinge. Each piston contains a rigid bottom electrode fabricated on the substrate and a compliant top electrode for capacitive transduction. 

In Kuntzman’s work [36], shown in Figure 7b,c, they sealed each capacitive structure under a vacuum. The coupling beam is designed to be stiff in bending while offering ideally zero stiffness against rotation about its pivot. Such a design addressed the dominant dissipation issue caused by the backplate structure in conventional capacitance microphones throughout audio frequencies. In Miles’s work [37], capacitive structure is enabled by using interdigitated comb fins, and the garnered results show that compared with existing dual-microphone systems (dual-membrane and dual backplate), the microphone aforementioned achieves a substantial improvement in sound pressure reference noise performance at low frequency bands.

However, it is also noteworthy that the teetertotter-style microphone currently offers only a conceptual approach for adjusting the stiffness of audio microphones. Its advantages predominantly lie in lower noise floors at low frequencies and the filtering nature of frequency response, rather than providing exceptional sensitivity or a flat frequency response. This type of microphone exclusively measures pressure gradients and, owing to its compact size, is constrained within frequencies above the audio bandwidth.

While directivity is not the primary motivation but rather an additional aspect of the device structure, further discussions in this regard will be expanded upon in the subsequent section.

The designs and performance parameters mentioned above have been presented in Table 1 for a more comprehensive comparison and summarization.

### 2.2. Piezoelectrical Transducer

**Figure 8:**
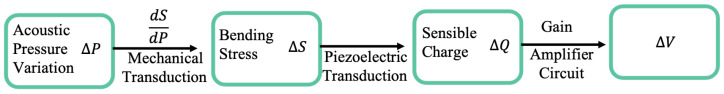
Working principle of the proposed piezoelectric MEMS microphone.

The working principle of the piezoelectrical microphone is illustrated in Figure 8. In essence, a piezoelectric microphone consists of a mechanical structure capable of converting the acoustic vibration into mechanical strain and thereafter a piezoelectric material that transforms this strain into electrical energy.

Given that an in-plane bending stress (or out-of-plane stress) ∆*S* is introduced on the cantilever (or membrane) in response to an applied sound pressure ∆*P*, and assuming a case of small piezoelectric coupling (SPC) in the MEMS microphone design, the sensing mechanism could be simplified as

(7) $$D=d_{xy\;}∆S,$$

(8) ∆Q=∫DdA,

where *D* is electrical displacement and ∆*S* refers to strain, while *d_xy_* denotes the electrical coefficient where *xy* can be 31 or 33 depending on the polarization direction of piezoelectric material, and A stands for the electrode area. As previously indicated, this review primarily focuses on advanced technology to enhance mechanical transduction, particularly in the first transduction stage within Figure 8, which is to increase the bending stress induced by the sound pressure. As a result, a substantial portion of research in this domain concentrates on structural design to maximize Δ*S* but within limited areas and while adjusting electrode configurations to achieve optimal transduction efficiency.

#### 2.2.1. The Reduction of Residual Stress

Piezoelectric MEMS microphones have attracted widespread attention due to the feature of passive devices (no power consumption), along with being quickly response, waterproof, and dustproof. However, the drawbacks of piezoelectric technology are apparent, where the primary issue is the compatibility with CMOS processes, and the dielectric losses of the piezoelectric material itself are considered the dominant source of noise compared to the input-referred noise of typical field-effect transistors and operational amplifiers [39]. The minimum detectable pressure is determined by the total input-referred noise integrated over a bandwidth of interest for a MEMS microphone [40], which is vital for performance in vocal applications. As an exemplified configuration, in which a center circular electrode with radius *r*_1_, a ring electrode with inner radius *r*_2_ and an outer radius rt that coincides with the total diaphragm radius, the MDP can be expressed as [41].

(9) $$MDP={32D}_{\;}\tilde{k}\sqrt{\frac{k_{b}T\tan\delta }{\varepsilon_{33}\pi \omega t^{3}}}\frac{1}{{r_{2}}^{2}\sqrt{{r_{1}}^{2}-{r_{2}}^{2}}},$$

(10) $$\tilde{k}=\frac{\varepsilon_{33}S_{11}^{E}\left(1-\upsilon \right)-2d_{31}^{E}}{d_{31}},$$

where *k_b_* is the Boltzmann constant, tan *δ* is the loss tangent of the material, *ω* is the frequency, *v* is the Poisson ratio, *ε*_33_ is the dielectric constant, *s*_11_ is the compliance, *d*_31_ is the piezoelectric coefficient, and *D* is the flexural rigidity of the layered diaphragm. Therefore, a strategic arrangement of electrodes is crucial to control the noise and enhance the sensitivity. Ullmann’s team [42] achieved an increase in output voltage by employing arranged segmented electrodes and utilizing flipped electrical polarity caused by opposite curvature over the membrane(two electrode) regions. Simultaneously they considered tensile stress and proposed the existence of a “sweet spot” in targeting the optimal design parameters to attain maximum output signal.

**Figure 9:**
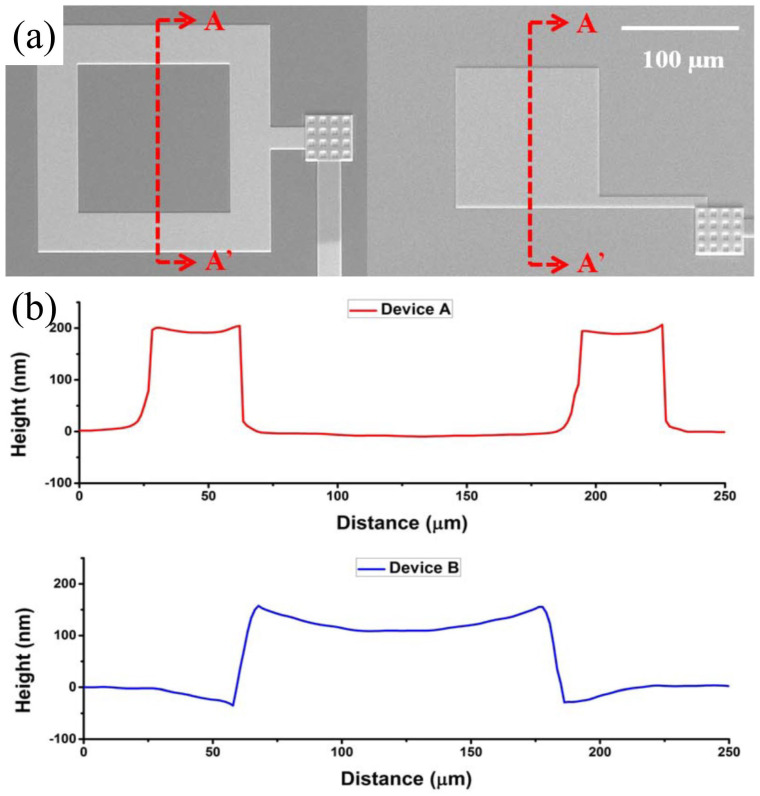
(**a**) SEM of the frame-like piezoelectric device. (**b**) 3-D images captured by holographic MEMS analyzer (Reprinted with permission from Ref. [45]).

Another issue faced by piezoelectric microphones, and so for all membrane-based structures, is the residual stress after fabrication, which can significantly impact the sensitivity of microphones [43]. Figure 9 shows the extracted surface profiles of the maximum deflection of a piezoelectric device; the deflection of a 250 μm membrane is 96 μm. A few trials have been conducted to address such an issue, including removing the buried oxide layer to reduce the membrane buckling or adding film coating in elastic material to tune the stiffness and improve the warpage of the sensing element [44].

Muralt et al. [46] optimized the fabrication process and exploited a compressively stressed oxide layer to balance the lead zirconate titanate (PZT) layer with tensile stress. Wang et al. [45] leveraged stress-free AlN thin films, a frame-like top electrode layout, and an integrated vacuum cavity to reduce initial stress and consequently achieve remarkable sensitivity at the resonant frequency.

Currently, limitations persist in the study of internal stress within manufacturing processes due to significant variations among manufacturing and relatively heavy reliance on empirical knowledge. The research focus on process-related internal stress is primarily geared towards addressing specific issues, therefore lacking the generation of universally applicable solutions.

The predominant approach to alleviating residual stress involves employing structures like slits or slit-like cantilevers; however, in turn, these may elevate the noise floor and compromise sensitivity, especially in low-frequency regimes [47]. Additionally, thin film residual stress will still be detrimental to cantilever diaphragms by bending the beam and enlarging the gap between the cantilever diaphragms, consequently reducing the acoustic resistance.

#### 2.2.2. Cantilever 

**Figure 10:**
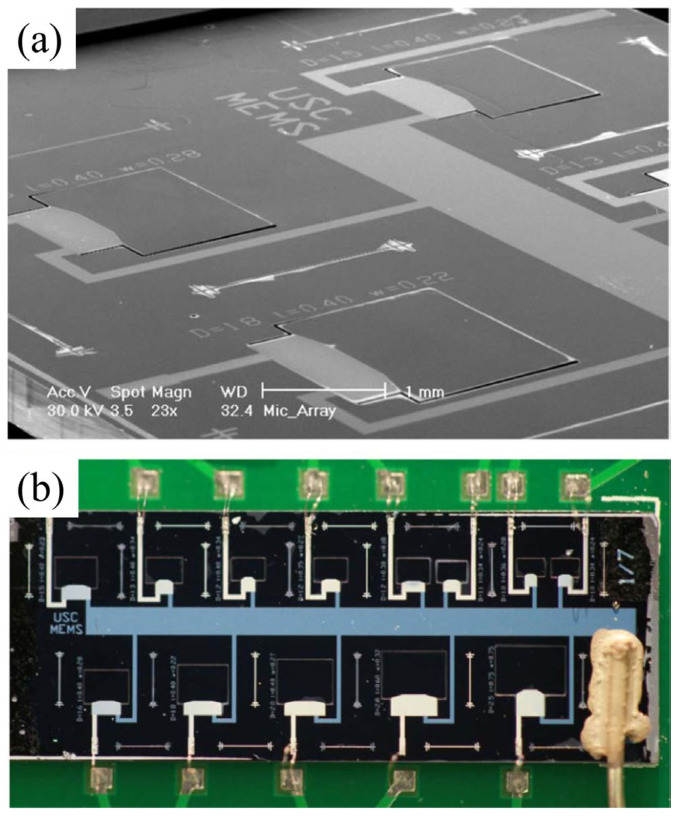
(**a**) SEM image of single unit (**b**) and complete array (Reprinted with permission from Ref. [49]).

In general, cantilevers are deployed in the form of arrays while a common electrode is shared for connecting to the readout circuit. Littrell et al. [48] proposed a cantilever array with a multi-layer of AlN. They presented closed-form expressions for the voltage developed across the structure. Baumgartel [49] proposed a cantilever array structure and demonstrated its application in signature detection characteristics, particularly in high levels of acoustic interference environments [50]. In this design, shown in Figure 10a,b each cantilever operates at a distinct resonant frequency and the overlap of these resonances effectively enhances the device sensitivity. Further, every single unit can also be used as an acoustic filter. However, the flaw lies in the uniformity of the gain, where a flat response that is akin to a conventional cantilever array is somewhat difficult to obtain. Moreover, the crosstalk should be well considered for practical utilization.

**Figure 11:**
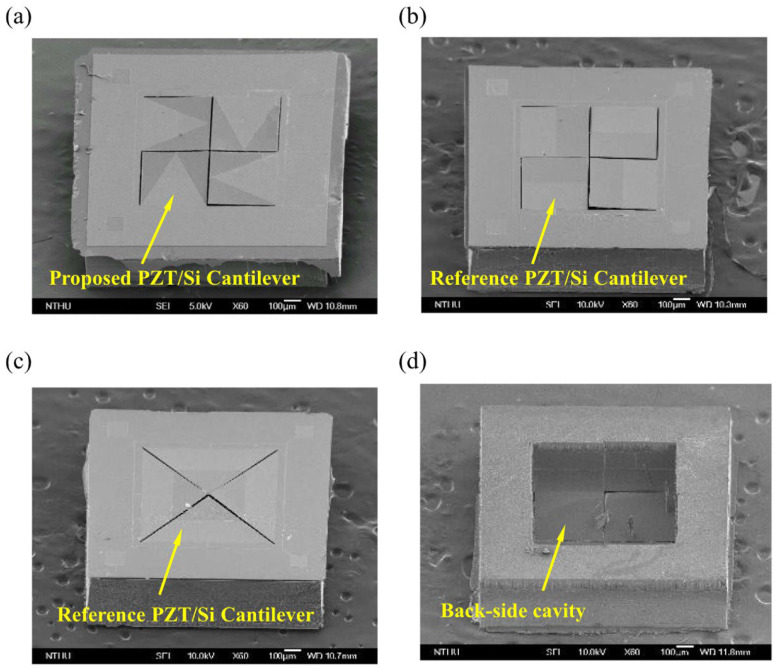
The scanning electron microscope (SEM) micrographs of typical fabricated piezoelectric MEMS microphone arrays (**a**) the rectangular Si cantilever with patterned PZT film. (**b**) the PZT/Si rectangular cantilever. (**c**) PZT/Si triangular cantilever (**d**) the back-side cavity of the microphone defined by the DRIE process (Reprinted with permission from Ref. [52]).

Apart from those aforementioned cantilever structures, scholars have explored the creation of arrays by slicing membranes into pieces, thereby achieving greater compliance. Chen’s group extensively investigated this technique and conducted a series of studies. Initially, they enhanced the sensitivity of the boundary and structure design of the triangular cantilever by two-stage etching and incorporating a trench in the sensing area [51]. Consecutively, they proposed a sliced square cantilever structure [52] to achieve higher stress and wider stress distribution than the 45-degree-right-triangle cantilever type as illustrated in Figure 11c which had been commercialized [53]. As illustrated in Figure 11b, they oriented the longitudinal axis of adjacent diaphragms orthogonally to reduce the gap between the bent cantilever diaphragms caused by residual stress, thence yielding higher SNR. Thereafter, in Figure 11a they further improved the design by partially removing the PZT layer (44% of the length of the cantilever) [54] to concentrate the stress on the PZT deposited area. Also, they came up with the idea of applying DC bias on the PZT layer to further reduce diaphragm bending. In the same year [55], they also conducted the work of partially removing the PZT and electrodes of the triangular shape cantilever array by adjusting the cross-sectional area of the piezoelectric layer and silicon, they manipulated the location of the neutral axis to increase the relative strain.

Hu and his team [56,57] introduced a cantilever structure featuring anchors positioned at the center of the working area of the device, demonstrating superior sensitivity compared to traditional array cantilevers with fixed boundaries using peripheral anchors. The cantilever, in this context, was shaped as a fan and an octagon, thereby promoting a relatively high signal-to-noise ratio (SNR) with the minimum possible diaphragm diameter.

Building upon previous work, Gong developed a novel two-AlN-layer tapered cantilever cluster PMUT with precise frequency control using layers [58]. They accomplished this by employing a specific PMUT release method that addressed residual stress through cantilever deformation while defining the cantilever boundary precisely via its front cavity. Across wafer frequency uniformity and relative deviation of the proposed design are 0.8% and 1% and meet the stringent 1% frequency control requirement for the first time.

The designs and performance parameters mentioned above have been presented in Table 2 for a more comprehensive comparison and summarization.

## 3. Denoising Techniques for High-Performance MEMS Microphones

With the advancement of microphone technology and the market, microphones have become carriers for various intelligent applications, therefore demanding higher criteria in noise suppression. For instance, a hearing aid should suppress ambient noise while delivering relevant sounds to the user [59]. Additionally, in specific environments, the noise performance of microphones is crucial for communication and intelligent voice-related needs such as voice activation and speech recognition.

### 3.1. Utilizing the Resonant Responses of Membranes

Due to inherent resonant responses in membrane devices, strategically configuring mechanical structures and quality factors (Q-factors) theoretically enables passive noise filtering and desired signal amplification. Reger et al. [60] implement such practices by detailing piezoelectric MEMS microphones leveraging aluminum nitride (AlN). They fine-tuned the resonant frequency by suspending a diaphragm using etched tethers anchored to the boundary. Inevitably mentioning that a flat frequency response of microphones is of great importance for accurately reproducing speech characteristics. Although distorted speech may not suit most speech recognition-based applications, the zero-power-consumption feature of the piezoelectric principles suggests that passive filters might find appropriate usage in certain wake-up applications.

In addition to the inherent resonance of thin films for filtering, acoustic resonators have been explored [61]. Kusano [62], among others, took inspiration from the human cochlea, employing a 3D-printed spiral-shaped structure. The structure is in conjunction with a microphone to filter and select a specific frequency range while suppressing others through resonance and anti-resonance frequencies. However, the attenuation level can significantly reduce the quality factor of the resonance, potentially limiting its suitability to specific application scenarios. Moreover, the relatively large size of the assembled device is adverse for applications aimed at miniaturized microphones.

### 3.2. Utilizing BF-Compliant Directional Microphones

Among the noise reduction schemes for MEMS microphones, beamforming has proven a highly effective technique. Such a method suppresses noise by weighting audio signals from various directions, particularly enhancing specific sound directions while suppressing others. Presently, beamforming primarily relies on omnidirectional microphone arrays, in which each output undergoes digital signal processing (DSP) techniques to manipulate specific time delays and phase adjustments. This necessitates an additional DSP module in the interface or ASIC circuitry. Furthermore, integrating microphone arrays into compact packages poses significant challenges. To tackle those problems, implementing noise suppression on the basis of the mechanical or sensor system design with directional selectivity would further drive device miniaturization. For instance, bi-directional sound sensors are able to achieve acoustic beamforming in practice and the directional characteristics can be easily changed according to the weighted sum of the signals acquired from only a pair of sensors [63].

As previously mentioned, whether through piezoelectric or capacitive transductions, teetertotter-style microphones inherently possess directional selectivity and can generate bi-polar directional patterns. This is attributed to both sound pressure intensity and sound pressure-gradient information, and can be described as

(11) $$p\left(x,t\right)\approx p\left(x,t\right)+x\frac{dp}{dx},$$

in which the first part and the second part of the equation describe the omnidirectional load and gradient load separately. The Oromia Ochracea-inspired teetertotter-style microphone that was initially proposed by Mills et al. [42,43,44,45,46], Refs. [64,65,66,67], enabled the creation of an eight-shaped polar pattern. Subsequent research involved adjustments in the relative sizes of the two wings [68], varying diaphragm thickness to modulate sensitivity [69], and integrating a force feedback setup to manage thermal–mechanical noise and active Q control [70]. For more comprehensive insights into this subject, additional relevant literature can be found in the review paper compiled by Ishfaque [7].

**Figure 12:**
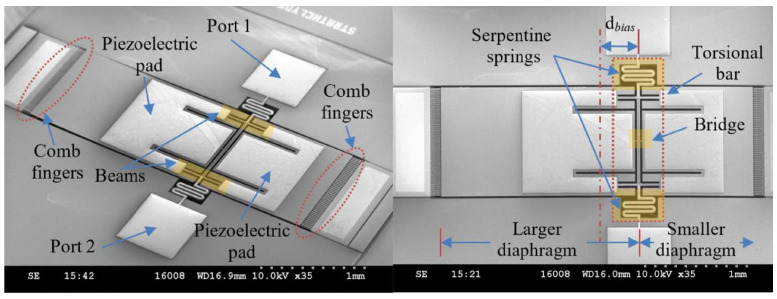
SEM images of the asymmetric microphone (Reprinted with permission from Ref. [72]).

The teetertotter-style microphone primarily operates within two resonant frequencies (two vibration modes) and their adjacent bands, thus restricting the sensor’s working bandwidth (usually <1 kHz). Considering the fact that signals below 1 kHz are crucial for speech applications and environmental noise localization [71]. As illustrated in Figure 12, Zhang et al. [72] achieved low-frequency applications at 500 Hz and 2 kHz by adjusting the central axis position of the device to modify resonant frequencies, and they utilized piezoelectric detection and capacitive auxiliary detection. Ren et al. further optimized Zhang’s work by tuning the two modal frequencies to 395 Hz and 739 Hz therefore leveraging the high vibration sensitivity of the fiber-optic Fabry-Perot interferometer (FPI) at the diaphragm’s distal edge [73]. These advancements aim to implement cost-effective miniature directional microphones with exceptional low-frequency Sound Source Localization (SSL) capability.

Whether operating with piezoelectric or capacitive transduction, the teetertotter-style microphone primarily faces challenges toward poor signal-to-noise ratio, narrow frequency bandwidth, and insufficiently flat responses [74]. Despite capacitive mechanism-based sensors having certain limitations in terms of device space compared to piezoelectric ones [37], they offer an alternative approach in terms of achieving a low-frequency sound pressure-referred noise floor and frequency selectivity. 

**Figure 13:**
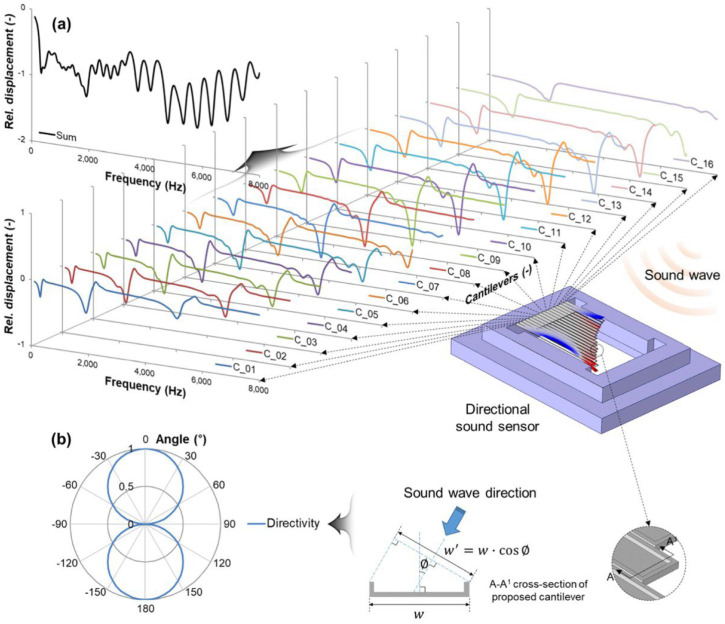
Design of proposed sensor. (**a**) Simulation model and results of cantilever displacements by a sound wave. (**b**) Bipolar directivity of proposed sensor (Reprinted with permission from Ref. [75]).

Inspired by the human cochlea, Kang et al. [75] proposed a bipolar (figure-of-8 pattern) directional sound sensor using 16 cantilevers operating under a resonant mode, as illustrated in Figure 13. Like the previous work of Baumgartel [49], these cantilevers have respective resonance frequencies and separately acquire signals to then combine them for sound sensing and cover a frequency range of 100 Hz to 8000 Hz, and overcome directional ambiguities introduced by bipolar directionality using a Canted Angle Design [63]. Another merit of cantilevers is the relatively low processing requirements since simple signal processing holds significant relevance for subsequent applications such as human voice localization and control design for wearable devices. However, in Kang’s work frequency-response ripples across for approximately 15 dB in the magnitude of sensitivity, occupying a significant proportion relative to the sensitivity data (−20 dB to −40 dB). Nonetheless, Kang’s work undoubtedly offers valuable insights for the subsequent design of directional microphones.

### 3.3. Other Applications in Noise Cancelation

**Figure 14:**
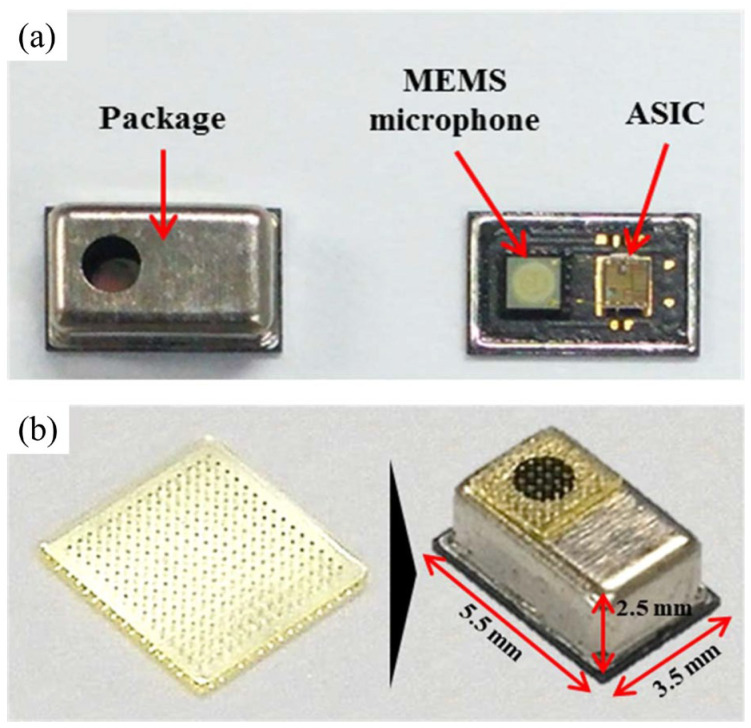
Directional MEMS microphone package: (**a**) Outside and inside images of package and (**b**) SU-8 filter attached on upper hole of package (Reprinted with permission from Ref. [30]).

For more specific applications, such as in-vehicle noise reduction, directional signal selection can also be achieved at the packaging level. As illustrated in Figure 14a, Yoo et al. [23] presented a unidirectional microphone that enables the suppression of noise signals from undesired directions. As illustrated in Figure 14b, The directional characteristic of the microphone is realized by attaching a porous SU-8 filter to facilitate a delay in one of the two acoustic ports on the package. Experimental data indicated the proposed unidirectional MEMS microphone along with the devised packaging shows a front-back ratio of 27.1 dB, resulting in an effective suppression of fixed-directional noise. However, its drawback compared to device-based design lies in the challenge of controlling directional selection through circuits. Additionally, Packaging level filtering does not necessitate a high manufacturing requirement for the sensor but high demands on the manufacturing for controlling the hole ratio of the filter and the packaging assembly.

Apart from suppressing specific directional noise, research also focuses on noise suppression in different frequency bands of omnidirectional microphones. Such designs primarily target applications akin to hearing aids. Although noises can be reduced using analog filters or digital signal processing [76] or a resonant microphone array (RMA), they cannot eliminate the original noises that directly get into the ear.

Hence, to address noise leakage, active noise cancellation (ANC) is carried out by picking up the noise in a specific frequency band [50]. Liu et al. [77] presented ANC based on MEMS RMA, and it demonstrated a better noise reduction level compared to flat band microphones. They used two sets of resonant microphone arrays composed of multiple piezoelectric cantilever microphones with different resonance frequencies covering two frequency ranges: one between 0.8 kHz to 5 kHz for vocal sensing and the other between 5 kHz to 9 kHz for ANC. The ANC was implemented with an analog inverter, digital phase compensator, digital adaptive filter, and deep learning technique, which outperformed the digital adaptive filter.

As the garnered results embodied, in all the tested cases, the word error rate improved with ANC, and the best performance was attained around the resonance frequencies of the resonant microphones. This suggests a way wherein specific frequencies’ active noise cancellation can be achieved through mechanical structural design.

## 4. Discussions and Conclusions

This article summarizes pivotal advancements in microphone development specifically for speech detection, which have been innovative and inspiring in recent years. To strengthen the technical comprehensiveness, this review provides a thorough interpretation of existing research concerning filtering and noise reduction performance.

Since its inception in 1980, this area has continued to capture research interests for decades and is poised to remain a focal point in the future. Referring to the prospective applications for microphones, the latent subjects include but are not limited to signal conversion in smartphones, wearable devices, applications in speech recognition, and voice localization.

As previously highlighted, microphones are products that involve a trade-off among multiple factors, including application scenarios, power consumption, manufacturing complexity, production cost, and environmental robustness. Therefore, to better adapt manifold applications focused on human speech, this article envisions the following for the practical design of future microphones:

- Presently, the primary bottleneck affecting microphone sensitivity and reliability remains within the manufacturing process. Stabilizing residual stress within thin film processing stands as a crucial means to significantly improve microphone performance and concurrently reduce production costs;
- The dynamic range of microphone devices presently hinges on both the mechanical structure and the noise floor. Thermal noise, circuit noise, and packaging noise collectively dictate the microphone noise performance. Integrating backend circuits with front-end sensors in a synergistic design approach will substantially diminish the overall noise and expand the available bandwidth;
- Sound source localization (SSL) technology predominantly relies on omnidirectional microphones. However, there is limited ongoing research on real-time sound source localization using directional microphones. Future developments should focus on merging MEMS multi-directional microphones with SSL algorithms to enable advanced applications like the real-time pinpointing of low-frequency noise and active noise cancellation.

## Figures and Tables

**Table 1:** The designs and performance parameters mentioned above are summarized.

Ref.	Chip Size	Sensitivity ^1^	Bandwidth	SNR
[38]	2 mm × 2 mm	−37.7 dBV/Pa	20 Hz–10 kHz	N/A
[14]	460 μm diameter *	−71 dBV/Pa **	300 Hz–20 kHz	N/A
[16]	512 μm diameter *	−68 dBV/Pa **	300 Hz–20 kHz	N/A
[17]	1.2 mm diameter *	−29.4 dBV/Pa	31 Hz–27 kHz	61.7 dB
[19]	5 mm × 4 mm	−38 dBFS/Pa	20 Hz–20 kHz	72 dB
[30]	300 μm diameter *	−64 dBV/Pa **	200 Hz–10 kHz	N/A
[23]	1.5 mm diameter *	−3.4 dBV/Pa	100 Hz–12 kHz	62.4 dB
[28]	1 mm × 1 mm	−40.5 dBV/Pa	50 Hz–20 kHz	57.8 dB
[29]	1.3 mm × 1.3 mm	−38 dBV/Pa	50 Hz–20 kHz	54 dB
[24]	1.2 mm diameter *	−60.1 dBV/Pa	1 kHz–20 kHz	N/A
[32]	0.3 mm × 0.3 mm *	−52 dBV/Pa	1 Hz–20 kHz	N/A
[12] ^4^	3.25 mm × 1.9 mm	−38 dBV/Pa	35 Hz–10 kHz	67 dB
[37]	1 mm × 3 mm *	−10.4 dBV/Pa ^3^	10 Hz–10 kHz	N/A
[36]	500 μm × 400 μm *	−111.3 dBV/Pa ^2^ **	20 Hz–250 kHz	N/A

* Size of membrane. ** Sensitivity unamplified. ^1^ Sensitivity is a reference to 1 V/Pa. ^2^ Sensitivity of mode 1. ^3^ Sensitivity at peak. ^4^ Acoustic over point is 134 dB SPL.

**Table 2:** The designs and performance parameters mentioned above are summarized.

Ref.	Chip Size	Sensitivity ^1^	Bandwidth	SNR
[42]	1/1.6 mm diameter *	−66.7 dBV/Pa	10 Hz–10 kHz	27/31 dB
[48]	30 μm × 369 μm ^2^	−79.1 dBV/Pa **	20 Hz–19 kHz	N/A
[49]	4 mm × 11 mm	−52 dBV/Pa	240 Hz–6.5 kHz	N/A
[51]	1080 μm × 1080 μm	−37.54 dBV/Pa	20 Hz–20 kHz	48.9 dB
[52]	800 μm × 800 μm	−35.9 dBV/Pa	100 Hz–10 kHz	70.1 dB
[54]	1.08 mm^2^	−32.1 dBV/Pa	1.4 Hz–10 kHz	77.2 dB
[55]	800 μm × 800 μm	−33.2 dBV/Pa	10 Hz–10 kHz	82.4 dB
[56]	800 μm diameter *	−73.7 dBV/Pa **	100 Hz–20 kHz	54.2 dB
[58]	170 μm diameter ^3^	−76.5 dBV/Pa **	20 Hz–6.3 kHz	N/A

* Size of membrane. ** Sensitivity unamplified. ^1^ Sensitivity is a reference to 1 V/Pa. ^2^ Size of the beam. ^3^ Size of the membrane.

## References

[1] Hohm D. An integrated silicon-electret-condenser microphone. Proceedings of the 11th International Congress on Acoustics.

[2] Wang L., Wang C., Wang Y., Quan A., Keshavarz M., Madeira B.P., Zhang H., Wang C., Kraft M. (2022). A review on coupled bulk acoustic wave mems resonators. Sensors.

[3] MEMS Microphones Market: Growth Potential of US$ 3.97 Billion. https://www.maximizemarketresearch.com/market-report/mems-microphones-market/186923/.

[4] Papila M., Haftka R.T., Nishida T., Sheplak M. (2006). Piezoresistive Microphone Design Pareto Optimization: Tradeoff between Sensitivity and Noise Floor. J. Microelectromechan. Syst..

[5] Pinto R.M.R., Gund V., Dias R.A., Nagaraja K.K., Vinayakumar K.B. (2022). CMOS-Integrated Aluminum Nitride MEMS: A Review. J. Microelectromechan. Syst..

[6] Fraga M.A., Furlan H., Pessoa R.S., Massi M. (2014). Wide bandgap semiconductor thin films for piezoelectric and piezoresistive MEMS sensors applied at high temperatures: An overview. Microsyst. Technol..

[7] Ishfaque A., Kim B. (2018). Fly Ormia Ochracea Inspired MEMS Directional Microphone: A Review. IEEE Sens. J..

[8] Shah M.A., Shah I.A., Lee D.-G., Hur S. (2019). Design approaches of MEMS microphones for enhanced performance. J. Sens..

[9] Zawawi S.A., Hamzah A.A., Majlis B.Y., Mohd-Yasin F. (2020). A Review of MEMS Capacitive Microphones. Micromachines.

[10] Kumar A., Varghese A., Sharma A., Prasad M., Janyani V., Yadav R., Elgaid K. (2022). Recent development and futuristic applications of MEMS based piezoelectric microphones. Sens. Actuators A Phys..

[11] Gemelli A., Tambussi M., Fusetto S., Aprile A., Moisello E., Bonizzoni E., Malcovati P. (2023). Recent Trends in Structures and Interfaces of MEMS Transducers for Audio Applications: A Review. Micromachines.

[12] Shubham S., Seo Y., Naderyan V., Song X., Frank A.J., Johnson J.T.M.G., da Silva M., Pedersen M. (2021). A Novel MEMS Capacitive Microphone with Semiconstrained Diaphragm Supported with Center and Peripheral Backplate Protrusions. Micromachines.

[13] Füldner M., Dehé A. Dual back plate silicon MEMS microphone: Balancing high performance. Proceedings of the DAGA 2015.

[14] Martin D., Kadirvel K., Liu J., Fox R., Sheplak M., Nishida T. Surface and bulk micromachined dual back-plate condenser microphone. Proceedings of the 18th IEEE International Conference on Micro Electro Mechanical Systems, 2005. MEMS 2005.

[15] Kadirvel K., Martin D.T., Liu J., Fox R., Sheplak M., Cattafesta L.N., Nishida T. Design, Modeling and Simulation of a Closed-Loop Controller for a Dual Backplate MEMS Capacitive Microphone. Proceedings of the 2007 IEEE Sensors.

[16] Martin D.T., Liu J., Kadirvel K., Fox R.M., Sheplak M., Nishida T. (2007). A Micromachined Dual-Backplate Capacitive Microphone for Aeroacoustic Measurements. J. Microelectromechan. Syst..

[17] Peña-García N.N., Aguilera-Cortés L.A., González-Palacios M.A., Raskin J.-P., Herrera-May A.L. (2018). Design and Modeling of a MEMS Dual-Backplate Capacitive Microphone with Spring-Supported Diaphragm for Mobile Device Applications. Sensors.

[18] Saadatmand M., Kook J. (2019). Multi-objective optimization of a circular dual back-plate MEMS microphone: Tradeoff between pull-in voltage, sensitivity and resonance frequency. Microsyst. Technol..

[19] Sant L., Fuldner M., Bach E., Conzatti F., Caspani A., Gaggl R., Baschirotto A., Wiesbauer A. (2022). A 130dB SPL 72dB SNR MEMS Microphone Using a Sealed-Dual Membrane Transducer and a Power-Scaling Read-Out ASIC. IEEE Sens. J..

[20] Klein W., Angelopoulos E., Barzen S., Fueldner M., Geissler S., Herrmann M.F., Krumbein U., Tkachuk K., Tosolini G., Wagner J. (2021). Membrane Support for Dual Backplate Transducers. U.S. Patent.

[21] Shubham S., Guo J. (2023). Dual-Diaphragm MEMS Transducers with High Effective Area. U.S. Patent.

[22] Scheeper P., Olthuis W., Bergveld P. (1994). The design, fabrication, and testing of corrugated silicon nitride diaphragms. J. Microelectromechan. Syst..

[23] Yoo I., Kim H., Yang S., Kim D., Kwon D.-S., Lee J., Jeong T. (2022). Development of Directional MEMS Microphone Single Module for High Directivity and SNR. IEEE Sens. J..

[24] Lo S.-C., Lai W.-C., Chang C.-I., Lo Y.-Y., Wang C., Bai M.R., Fang W. Development of a no-back-plate SOI MEMS condenser microphone. Proceedings of the 2015 Transducers—2015 18th International Solid-State Sensors, Actuators and Microsystems Conference.

[25] Jerman J. (1990). The fabrication and use of micromachined corrugated silicon diaphragms. Sens. Actuators A Phys..

[26] Spiering V.L., Bouwstra S., Spiering R.M. (1993). On-chip decoupling zone for package-stress reduction. Sens. Actuators A Phys..

[27] Dehe A., Barzen S., Friza W., Klein W. (2019). Semiconductor Devices Having a Membrane Layer with Smooth Stress-Relieving Corrugations and Methods of Fabrication Thereof. U.S. Patent.

[28] Lo S.-C., Chan C.-K., Lee Y.-C., Wu M., Fang W. Implementation of Two-Poly Differential MEMS Microphones for SNR and Sensing Range Enhancement. Proceedings of the 2019 IEEE 32nd International Conference on Micro Electro Mechanical Systems (MEMS).

[29] Lo S.-C., Yeh S.-K., Wang J.-J., Wu M., Chen R., Fang W. Bandwidth and SNR enhancement of MEMS microphones using two poly-Si micromachining processes. Proceedings of the 2018 IEEE Micro Electro Mechanical Systems (MEMS).

[30] Mao W.-J., Cheng C.-L., Lo S.-C., Chen Y.-S., Fang W. Design and implementation of a CMOS-MEMS microphone without the back-plate. Proceedings of the 2017 19th International Conference on Solid-State Sensors, Actuators and Microsystems (TRANSDUCERS).

[31] Ganji B.A., Sedaghat S.B., Roncaglia A., Belsito L. (2018). Design and fabrication of very small MEMS microphone with silicon diaphragm supported by Z-shape arms using SOI wafer. Solid-State Electron..

[32] Weigold J., Brosnihan T., Bergeron J., Zhang X. A MEMS Condenser Microphone for Consumer Applications. Proceedings of the 19th IEEE International Conference on Micro Electro Mechanical Systems.

[33] Loeppert P.V. (2022). Mems Structure with Stiffening Member. U.S. Patent.

[34] Touse M., Sinibaldi J., Simsek K., Catterlin J., Harrison S., Karunasiri G. (2010). Fabrication of a microelectromechanical directional sound sensor with electronic readout using comb fingers. Appl. Phys. Lett..

[35] Liu H., Currano L., Gee D., Helms T., Yu M. (2013). Understanding and mimicking the dual optimality of the fly ear. Sci. Rep..

[36] Kuntzman M.L., Kim D., Hall N.A. (2015). Microfabrication and Experimental Evaluation of a Rotational Capacitive Micromachined Ultrasonic Transducer. J. Microelectromechan. Syst..

[37] Miles R.N., Cui W., Su Q.T., Homentcovschi D. (2015). A MEMS Low-Noise Sound Pressure Gradient Microphone with Capacitive Sensing. J. Microelectromechan. Syst..

[38] Rombach P., Müllenborn M., Klein U., Rasmussen K. (2002). The first low voltage, low noise differential silicon microphone, technology development and measurement results. Sens. Actuators A Phys..

[39] Hake A.E., Zhao C., Ping L., Grosh K. (2020). Ultraminiature AlN diaphragm acoustic transducer. Appl. Phys. Lett..

[40] Williams M.D., Griffin B.A., Reagan T.N., Underbrink J.R., Sheplak M. (2012). An AlN MEMS Piezoelectric Microphone for Aeroacoustic Applications. J. Microelectromechan. Syst..

[41] Littrell R.J. (2010). High Performance Piezoelectric MEMS Microphones. Ph.D. Thesis.

[42] Ullmann P., Bretthauer C., Schneider M., Schmid U. (2023). Stress analysis of circular membrane-type MEMS microphones with piezoelectric read-out. Sens. Actuators A Phys..

[43] Lee S.S., Ried R.P., White R.M. (1996). Piezoelectric cantilever microphone and microspeaker. J. Microelectromechan. Syst..

[44] Kuchiji H., Masumoto N., Baba A. (2023). Piezoelectric MEMS wideband acoustic sensor coated by organic film. Jpn. J. Appl. Phys..

[45] Wang T., Lee C. (2015). Zero-Bending Piezoelectric Micromachined Ultrasonic Transducer (pMUT) with Enhanced Transmitting Performance. J. Microelectromechan. Syst..

[46] Muralt P., Ledermann N., Baborowski J., Barzegar A., Gentil S., Belgacem B., Petitgrand S., Bosseboeuf A., Setter N. (2005). Piezoelectric micromachined ultrasonic transducers based on PZT thin films. IEEE Trans. Ultrason. Ferroelectr. Freq. Control.

[47] Kuenzig T., Schrag G., Dehe A., Wachutka G. Performance and noise analysis of capacitive silicon microphones using tailored system-level simulation. Proceedings of the 2015 Transducers-2015 18th International Conference on Solid-State Sensors, Actuators and Microsystems (TRANSDUCERS).

[48] Littrell R., Grosh K. (2012). Modeling and Characterization of Cantilever-Based MEMS Piezoelectric Sensors and Actuators. J. Microelectromechan. Syst..

[49] Baumgartel L., Vafanejad A., Chen S.-J., Kim E.S. (2013). Resonance-Enhanced Piezoelectric Microphone Array for Broadband or Prefiltered Acoustic Sensing. J. Microelectromechan. Syst..

[50] Shkel A.A., Baumgartel L., Kim E.S. A resonant piezoelectric microphone array for detection of acoustic signatures in noisy environments. Proceedings of the 2015 28th IEEE International Conference on Micro Electro Mechanical Systems (MEMS).

[51] Tseng S.-H., Lo S.-C., Chen Y.-C., Lee Y.-C., Wu M., Fang W. Implementation of Piezoelectric MEMS Microphone for Sensitivity and Sensing Range Enhancement. Proceedings of the 2020 IEEE 33rd International Conference on Micro Electro Mechanical Systems (MEMS).

[52] Chen Y.-C., Lo S.-C., Cheng H.-H., Wu M., Huang I.-Y., Fang W. Design of Cantilever Diaphragm Array Piezoelectric MEMS Microphone for Signal-To-Noise Ratio Enhancement. Proceedings of the 2019 IEEE Sensors.

[53] Ledermann N., Muralt P., Baborowski J., Forster M., Pellaux J.-P. (2004). Piezoelectric Pb(Zr*_x_*, Ti_1−*x*_)O_3_ thin film cantilever and bridge acoustic sensors for miniaturized photoacoustic gas detectors. J. Micromechan. Microeng..

[54] Wang S.-D., Chen Y.-C., Lo S.-C., Wang Y.-J., Wu M., Fang W. On The Performance Enhancement of Cantilever Diaphragm Piezoelectric Microphone. Proceedings of the 2021 IEEE Sensors.

[55] Chen Y.-C., Lo S.-C., Wang S.-D., Wang Y.-J., Wu M., Fang W. (2021). On the PZT/Si unimorph cantilever design for the signal-to-noise ratio enhancement of piezoelectric MEMS microphone. J. Micromechan. Microeng..

[56] Hu B., Liu W., Yang C., Lu L., Wang Z., Liu Y., Cai Y., Wang J., Guo S., Sun C. (2023). A ScAlN-Based Piezoelectric MEMS Microphone with Sector-Connected Cantilevers. J. Microelectromechan. Syst..

[57] Yang C., Hu B., Lu L., Wang Y., Cai Y., Liu Y., Liu W., Sun C. Bimorph Piezoelectric MEMS Microphone with Tractive Structure. Proceedings of the 2022 IEEE International Ultrasonics Symposium (IUS).

[58] Gong Y., Zhang M., Sun S., Guo W., Sun C., Pang W. (2023). Piezoelectric Micromachined Ultrasonic Transducers with Superior Frequency Control. J. Microelectromechan. Syst..

[59] Ho C.-Y., Shyu K.-K., Chang C.-Y., Kuo S.M. (2018). Integrated active noise control for open-fit hearing aids with customized filter. Appl. Acoust..

[60] Reger R.W., Clews P.J., Bryan G.M., Keane C.A., Henry M.D., Griffin B.A. Aluminum nitride piezoelectric microphones as zero-power passive acoustic filters. Proceedings of the 2017 19th International Conference on Solid-State Sensors, Actuators and Microsystems (TRANSDUCERS).

[61] Cai X., Guo Q., Hu G., Yang J. (2014). Ultrathin low-frequency sound absorbing panels based on coplanar spiral tubes or coplanar Helmholtz resonators. Appl. Phys. Lett..

[62] Kusano Y., Segovia-Fernandez J., Sonmezoglu S., Amirtharajah R., Horsley D.A. Frequency selective mems microphone based on a bioinspired spiral-shaped acoustic resonator. Proceedings of the 2017 19th International Conference on Solid-State Sensors, Actuators and Microsystems (TRANSDUCERS).

[63] Wilmott D., Alves F., Karunasiri G. (2016). Bio-Inspired Miniature Direction Finding Acoustic Sensor. Sci. Rep..

[64] Miles R.N., Robert D., Hoy R.R. (1995). Mechanically coupled ears for directional hearing in the parasitoid fly *Ormia ochracea*. J. Acoust. Soc. Am..

[65] Miles R.N., Su Q., Cui W., Shetye M., Degertekin F.L., Bicen B., Garcia C., Jones S., Hall N. (2009). A low-noise differential microphone inspired by the ears of the parasitoid fly *Ormia ochracea*. J. Acoust. Soc. Am..

[66] Miles R.N., Gibbons C., Gao J., Yoo K., Su Q., Cui W. (2001). A silicon nitride microphone diaphragm inspired by the ears of the parasitoid fly *Ormia ochracea*. J. Acoust. Soc. Am..

[67] Tan L., Miles R.N., Weinstein M.G., Miller R.A., Su Q., Cui W., Gao J. (2002). Response of a biologically inspired MEMS differential microphone diaphragm. Unattended Ground Sensor Technologies and Applications IV.

[68] Touse M., Sinibaldi J., Karunasiri G. MEMS directional sound sensor with simultaneous detection of two frequency bands. Proceedings of the 2010 Ninth IEEE Sensors Conference (SENSORS 2010).

[69] Downey R.H., Karunasiri G. (2013). Reduced residual stress curvature and branched comb fingers increase sensitivity of MEMS acoustic sensor. J. Microelectromechan. Syst..

[70] Bicen B. (2010). Micromachined Diffraction Based Optical Microphones and Intensity Probes with Electrostatic Force Feedback. Ph.D. Thesis.

[71] Ang L.Y.L., Koh Y.K., Lee H.P. (2019). Plate-type acoustic metamaterials: Experimental evaluation of a modular large-scale design for low-frequency noise con-trol. Acoustics.

[72] Zhang Y., Bauer R., Jackson J.C., Whitmer W.M., Windmill J.F.C., Uttamchandani D. (2018). A Low-Frequency Dual-Band Operational Microphone Mimicking the Hearing Property of *Ormia Ochracea*. J. Microelectromechan. Syst..

[73] Ren D., Liu X., Zhang M., Gao R., Qi Z.-M. (2021). Low-Frequency Bi-Directional Microphone Based on a Combination of Bionic MEMS Diaphragm and Fiber Acousto-Optic Transducer. IEEE Sens. J..

[74] Baumhauer J.C., Fengyuan L., Marcus L.A., Michel A.D., Reese M. (2018). Gradient Micro-Electro-Mechanical Systems (MEMS) Microphone. U.S. Patent.

[75] Kang S., Hong H.-K., Rhee C.-H., Yoon Y., Kim C.-H. (2021). Directional Sound Sensor with Consistent Directivity and Sensitivity in the Audible Range. J. Microelectromechan. Syst..

[76] Chung K. (2004). Challenges and Recent Developments in Hearing Aids: Part II. Feedback and Occlusion Effect Reduction Strategies, Laser Shell Manufacturing Processes, and Other Signal Processing Technologies. Trends Amplif..

[77] Liu H., Liu S., Shkel A.A., Kim E.S. (2020). Active Noise Cancellation with MEMS Resonant Microphone Array. J. Microelectromechan. Syst..

